# Untargeted Serum Proteomics in the Fontan Circulation Reveals Three Distinct Molecular Signatures of Fontan Physiology with CYB5R3 Among Key Proteins

**DOI:** 10.3390/ijms27031220

**Published:** 2026-01-26

**Authors:** Alexander Blaha, David Renaud, Fatima Ageed, Bettina Sarg, Klaus Faserl, Alexander Kirchmair, Dietmar Rieder, Isabel Mihajlovic, Nele Ströbel, Kai Thorsten Laser, Miriam Michel

**Affiliations:** 1 Department of Child and Adolescent Health, Division of Pediatrics III—Cardiology, Pulmonology, Allergology and Cystic Fibrosis, Medical University of Innsbruck, 6020 Innsbruck, Austria; alexander.blaha@student.i-med.ac.at (A.B.); david.renaud.chd@gmail.com (D.R.); fatimaageed88@gmail.com (F.A.); isabel.mihajlovic@student.i-med.ac.at (I.M.); nele.stroebel@student.i-med.ac.at (N.S.); 2 Biocenter, Protein Core Facility, Medical University of Innsbruck, 6020 Innsbruck, Austria; bettina.sarg@i-med.ac.at (B.S.); klaus.faserl@i-med.ac.at (K.F.); 3 Biocenter, Bioinformatics Core Facility, Medical University of Innsbruck, 6020 Innsbruck, Austria; alexander.kirchmair@i-med.ac.at; 4 Biocenter, Institute of Bioinformatics, Medical University of Innsbruck, 6020 Innsbruck, Austria; dietmar.rieder@i-med.ac.at; 5 Department of Pediatrics and Adolescent Medicine, University Hospital of Giessen and Marburg, 35392 Giessen, Germany; kaithorsten.laser@uk-gm.de

**Keywords:** congenital heart disease, Fontan, proteomics, CYB5R3, biomarkers, mass spectrometry, risk stratification, single ventricle

## Abstract

The total cavopulmonary anastomosis (Fontan procedure), a palliative procedure for single-ventricle congenital heart disease, improves survival but is associated with progressive multiorgan complications and high long-term morbidity. Prior blood-based proteomic studies in adults have been limited to targeted antibody-based panels or focused on methodological comparisons. Systemic molecular alterations in younger, clinically heterogeneous patients, particularly in untargeted pathways, remain incompletely characterized. Serum samples from 48 Fontan patients and 48 age- and sex-matched healthy controls were analyzed using mass spectrometry with TMT labeling. 2228 proteins were quantified, of which 124 were significantly differentially abundant (fold change > 1.5 or <0.67, FDR-adjusted *p* < 0.05). Network analysis identified three major functional clusters: extracellular matrix (ECM) organization (predominantly increased), actin cytoskeleton organization, and platelet-related pathways (both predominantly decreased). Stratified analyses showed reduced ECM protein abundance in high-risk patients, suggesting a shift from active remodeling toward a more established fibrotic state, and uniquely elevated cytochrome b5 reductase 3 (CYB5R3), implicating altered redox homeostasis, nitric oxide metabolism, and cellular aging. Overall, our findings extend prior targeted analyses, reveal potential biomarkers such as CYB5R3 and underscore the complexity of the Fontan circulation, with implications for risk stratification and therapeutic targeting.

## 1. Introduction

Single-ventricle heart disease (SVHD) comprises complex congenital heart defects characterized by the presence of only one fully developed and functional heart chamber [1,2]. Patients typically undergo staged surgical palliation culminating in the Fontan circulation, in which the systemic venous return is directly routed to the pulmonary arteries, bypassing a subpulmonary ventricle [3]. Natural consequences include elevated central venous pressure, non-pulsatile blood flow, and limited systemic cardiac output. While survival rate has improved substantially, the Fontan circulation is associated with significant long-term morbidity, including heart failure, arrhythmias, thromboembolic events, liver disease, protein-losing enteropathy (PLE) [4], and metabolic disturbances [5].

To date, only a few blood-based proteomic studies have investigated Fontan-associated molecular alterations. Two studies in adult cohorts employed affinity-based plasma proteomics [6,7]. In a deliberately selected group of clinically stable, high-performing adult patients with a systemic left ventricle (LV), good exercise capacity, and no overt comorbidities, our group used an antibody microarray (Sciomics GmbH, Neckargemünd, Germany) to identify Fontan-specific signatures [6]. Kelly et al. subsequently applied an Olink^(R)^ platform in a larger and more heterogeneous adult cohort, revealing consistent alterations suggestive of a pro-inflammatory, pro-fibrotic, and pro-angiogenic state [7].

More recently, Assi et al. analyzed plasma proteomes in adults with a Fontan circulation using two high-throughput affinity-based platforms (Olink^(R)^ and Somascan^(R)^). In contrast to prior studies, the primary focus was a methodological comparison between antibody- and aptamer-based assays rather than biological pathway discovery. While partial overlap between platforms was observed, the authors emphasized substantial assay-dependent variability [8]. In addition, Lecointe et al. investigated plasma microvesicle-associated proteomes in a small pediatric cohort with advanced cardiac dysfunction using mass spectrometry. They identified signatures related to oxidative stress, mitochondrial dysfunction, and cell death, highlighting biological processes not captured by bulk plasma affinity-based approaches [9].

The present study extends this work by applying mass spectrometry-based serum proteomics to a younger and clinically heterogeneous Fontan population (with respect to ventricular morphology, exercise capacity, and comorbidities). This approach enables unbiased detection of a broader range of protein classes and molecular forms, providing complementary insight beyond affinity-based platforms and allowing the identification of proteomic patterns associated with distinct Fontan-related clinical phenotypes [6,7,9].

## 2. Results

The Fontan patients had a mean age of 18 years, with 44% female participants, comparable to the control group. On average, 14.9 years had passed since the Fontan operation, and nearly half of the patients presented with a morphologically RV. Several Fontan-specific risk factors were assessed, comprising 12 predefined clinical parameters, each contributing one point to a composite score, with >3 defining an unfavorable Fontan profile. The majority of patients (77%) had a composite risk score below 4, reflecting a cohort characterized by an overall more favorable Fontan profile. Furthermore, 90% of patients received anticoagulation with a vitamin K antagonist. Complete data were not available for all 48 patients, resulting in variations in sample size (n) across parameters. Table 1 provides participant characteristics and risk factors, including the 12 parameters that constitute the risk score (indicated by an asterix), while Supplementary Data S1 provides a more detailed overview.

### 2.1. Differential Proteomic Profile: Fontan Patients vs. Controls

**Figure 1:**
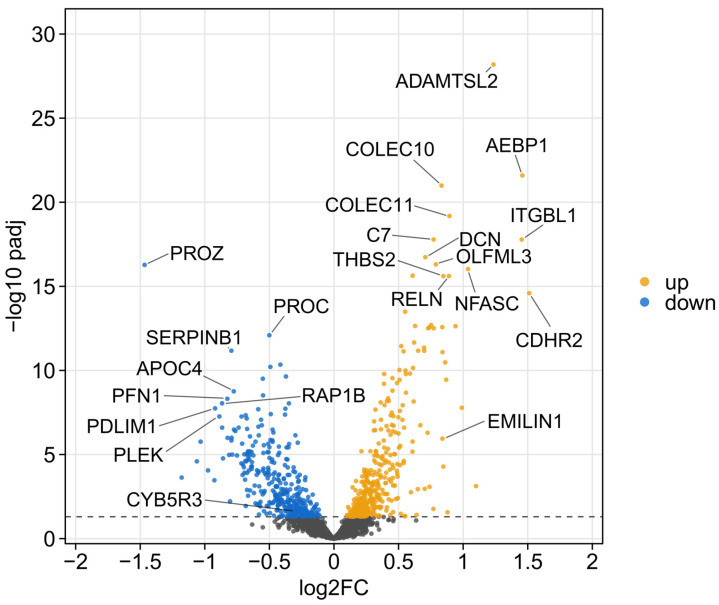
Volcano plot comparing Fontan patients and controls. Each dot represents a quantified protein. The x-axis shows the log2 fold change (log2FC) in protein abundance (Fontan vs. Control) and the y-axis displays the −log10 adjusted *p*-value (−log10 *p*_adj). Higher values indicate greater statistical significance. The dashed horizontal line denotes the significance threshold (adjusted *p* < 0.05). Proteins with significantly increased abundance are shown in yellow, whereas significantly decreased proteins are shown in blue. Selected highly significant proteins are labeled for clarity. Full protein identifiers are provided in Supplementary Data S2. (abbreviations according to UniProt [10]).

The comparison of Fontan patients with healthy controls revealed a broad pattern of differential protein abundance as visualized by the volcano plot (Figure 1). A substantial number of proteins deviated significantly from baseline levels, with 808 of them exceeding the threshold for statistical significance (adjusted *p*-value < 0.05). In the plot, proteins with higher abundance in patients with a Fontan circulation are shown in yellow, while those marked in blue indicate downregulation relative to controls.

### 2.2. Overview of Untargeted Proteomic Analysis and Network Interpretation

**Figure 2:**
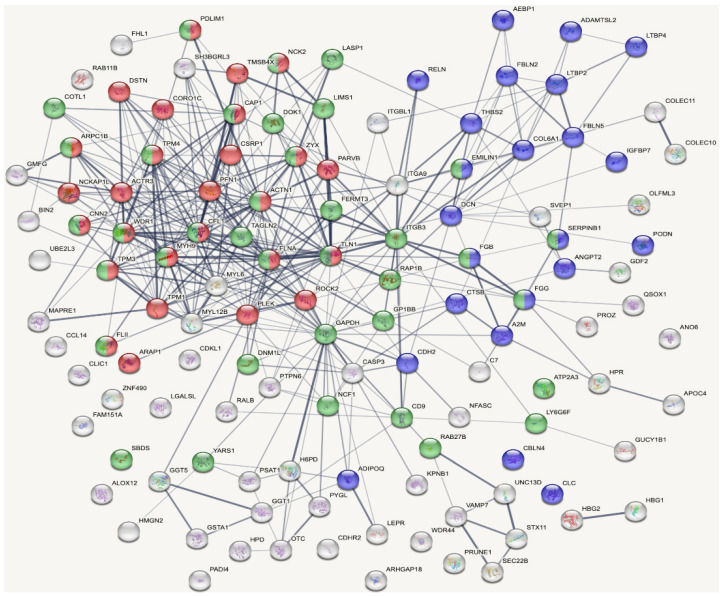
Protein–protein interaction network of differentially expressed proteins. The network is based on 124 differentially expressed proteins identified by cluster analysis using filtering criteria of abundance ratios greater than 1.5 or less than 0.67 and an adjusted *p*-value below 0.05. Each node represents a protein, and edges indicate known or predicted functional or physical associations. Edge thickness reflects the strength of supporting evidence. The network reveals three major functional clusters corresponding to extracellular matrix proteins shown in blue, actin cytoskeleton organization shown in red, and platelet-associated proteins shown in green. Since all proteins in the network are labeled, complete identifiers can be cross-checked in UniProt [10].

The untargeted mass spectrometry approach enabled the detection of 2288 distinct serum proteins across the entire study cohort (Supplementary Data S2). As seen in the global volcano plot comparing Fontan patients and healthy controls (Figure 1), many proteins showed statistically significant differences in abundance. To enable a more focused analysis, we applied filtering criteria based on abundance ratios (>1.5 or <0.67) and adjusted *p*-values (<0.05). This yielded a subset of 124 proteins considered for further biological interpretation. For network-based functional insight, these 124 proteins were analyzed using the STRING database, which enabled the construction of interaction maps based on known and predicted protein–protein associations (Figure 2).

The resulting network revealed three main functional clusters. The first cluster (23 proteins) was enriched in ECM organization and remodeling, the second (27 proteins) was related to actin cytoskeleton organization (ACO), and the third (37 proteins) to platelet-associated proteins (PAPs). ECM proteins were predominantly high in abundance, whereas proteins linked to actin dynamics and platelet function were largely low in abundance (Table 2).

### 2.3. Case Study: Platelet-Associated Proteins in a Non-Anticoagulated Fontan Patient

**Figure 3:**
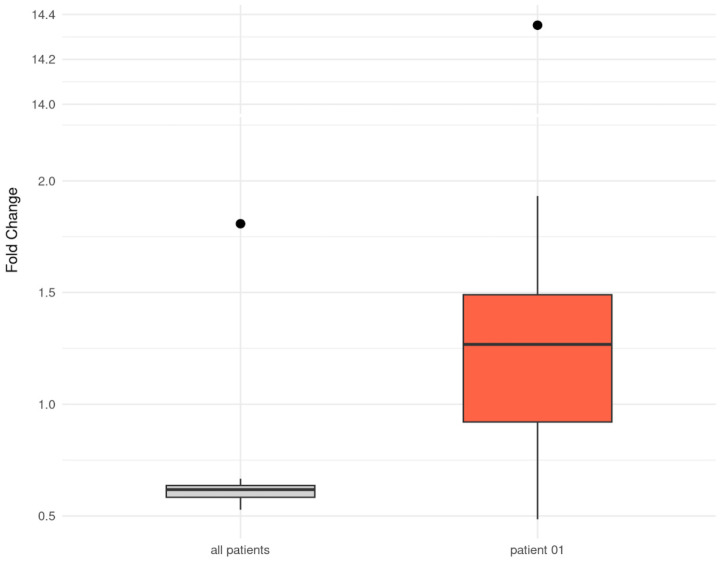
Boxplot: Comparison of fold change (FC) of the 37 platelet-associated proteins (PAPs) between all Fontan patients vs. controls and patient 01 vs. control 01. The grey boxplot shows the distribution of FC for the 37 PAPs in all Fontan patients receiving antithrombotic therapy. The red boxplot shows the distribution of the same 37 PAPs in patient 01, who was not receiving anticoagulant or antiplatelet medication. Patient 01 exhibits a distinct profile with generally higher protein levels compared to the cohort. EMILIN1 appears as a prominent outlier and is shown as a single data point above the axis break. In contrast, CNN2, LASP1, NCK2, DOK1, COTL1, TAGLN2, PDLIM1, SERPINB1, and LY6G6F remained downregulated in patient 01.

To further explore the PAPs cluster, we performed a focused single-patient analysis assessing the effect of absent antithrombotic therapy. Patient 01 was younger than the cohort mean (9 vs. 18.4 years), classified as having an unfavorable Fontan profile (risk score > 3), and had undergone TCPC 9.6 years previously (cohort mean: 14.9 years). Compared with a matched healthy control, only 9 of 37 platelet proteins remained reduced in abundance in the patient without anticoagulant or antiplatelet medication, while 34 of 37 were reduced in those receiving therapy. Notably, elastic microfibril interface located protein-1 (EMILIN1) showed a FC of 14.4 in patient 01 compared with the corresponding control (Figure 3).

### 2.4. Fontan Circulation Status

**Figure 4:**
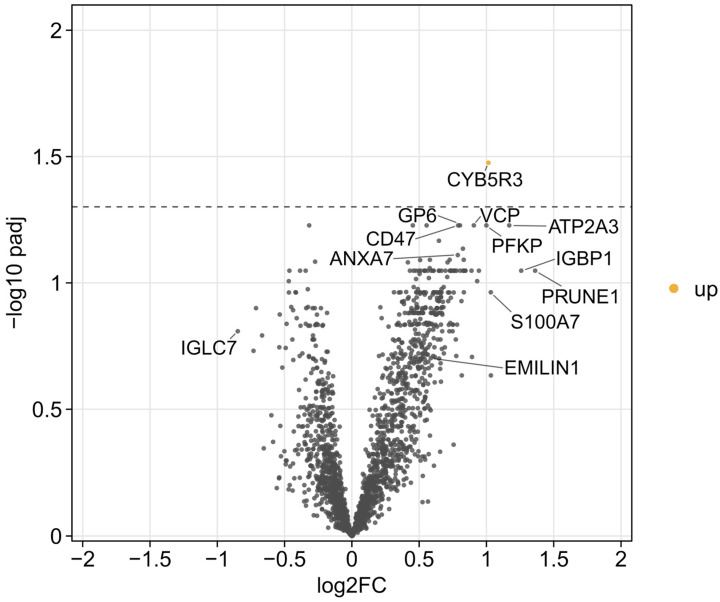
Volcano plot comparing patients with favorable and unfavorable Fontan profiles. Each dot represents a quantified protein. The x-axis shows the log2 fold change (unfavorable vs. favorable), and the y-axis displays the −log10 adjusted *p*-value. The dashed horizontal line indicates the significance threshold (adjusted *p* < 0.05). Significantly upregulated proteins are highlighted in yellow. Among all quantified proteins, only CYB5R3 exceeds the significance threshold.

In the subgroup analysis comparing patients categorized by a favorable (n = 37) and unfavorable Fontan profile (n = 11), cytochrome b5 reductase 3 (CYB5R3) was the only protein to reach the predefined threshold for statistical significance (Figure 4). CYB5R3 abundance was higher in the Fontan group with a risk score > 3. CYB5R3 is a redox enzyme involved in oxygen species regulation and endothelial function.

### 2.5. Gene Set Enrichment Analysis (GSEA)—Results of Selected Gene Sets

**Figure A1:**
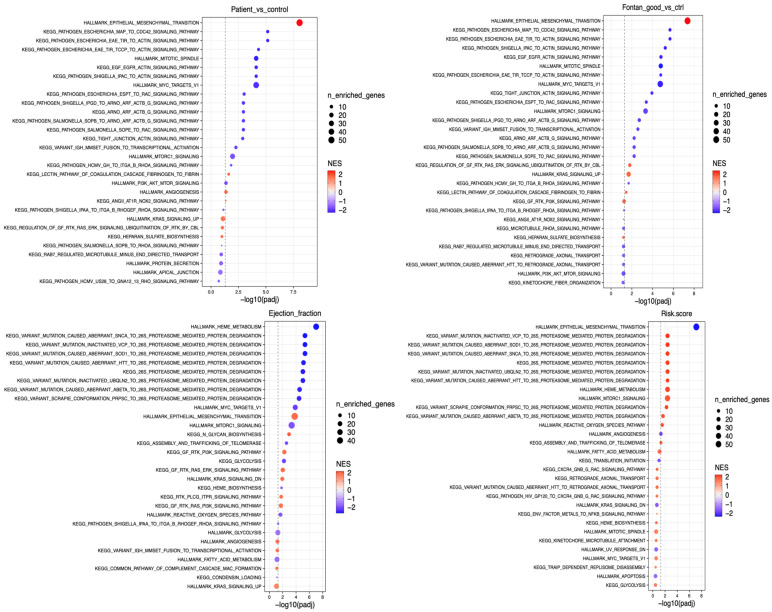
GSEA of Molecular Signatures Database Hallmark and KEGG gene sets. This analysis includes comparisons between Fontan patients and controls, as well as comparisons within patient subgroups based on ejection fraction (high vs. low) and risk score. The x-axis represents −log_10_ (FDR-adjusted *p*-value), dot size corresponds to the number of enriched genes per pathway, and color indicates the normalized enrichment score (NES).

**Figure A2:**
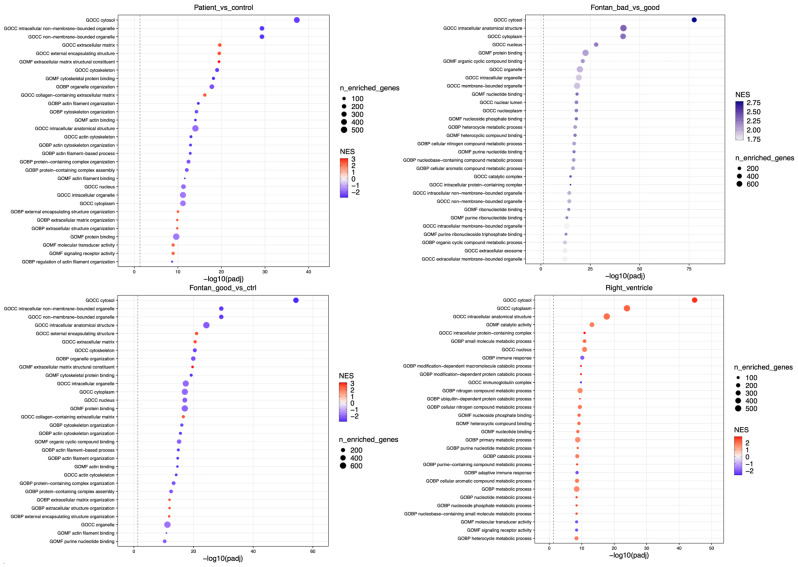
GSEA of Gene Ontology (GO) gene sets. Comparisons between Fontan patients and controls, as well as within patient subgroups (unfavorable vs. favorable Fontan and right ventricle morphology (yes vs. no)). The x-axis represents −log_10_ (FDR-adjusted *p*-value), dot size corresponds to the number of enriched genes per set, and color indicates the normalized enrichment score (NES).

For the hallmark epithelial–mesenchymal transition (HEMT) gene set, all comparisons involving the control group showed enrichment in Fontan patients, including the overall patient cohort and both favorable and unfavorable Fontan profile subgroups (all with positive normalized enrichment scores (NES) values). In contrast, subgroup comparisons within the Fontan cohort revealed negative NES values for patients with low EF (low, n = 23 vs. high, n = 25) and those classified as high-risk (risk score > 3 vs. ≤3), indicating reduced HEMT-related protein abundance in these subgroups (Figure A1). Conversely, the Gene Ontology Cellular Component (GOCC) cytosol gene set displayed an opposite trend. Here, NES values were lower in comparisons of the patient cohort with the controls but increased in subgroup analyses. Enrichment was observed in patients with an unfavorable Fontan profile (n = 11) when directly compared with those exhibiting a favorable profile (n = 37). In addition, patients with a systemic RV (n = 22) showed enrichment in NES compared with those with a systemic LV (n = 26) (Figure A2).

## 3. Discussion

By complementing findings from previous proteomic approaches in individuals with a Fontan circulation, this work further delineates systemic molecular alterations specific to Fontan physiology and its clinical phenotypes. In a younger and more clinically heterogeneous cohort, we confirm profound systemic proteomic remodeling, with ECM, ACO, and PAPs emerging as hallmarks of Fontan physiology. Our study expands the molecular scope beyond that captured by the previously published antibody-based panels [6,7], who investigated predominantly adult patients and identified markers mainly related to inflammation and angiogenesis. Mass spectrometry-based proteomics allows the detection of additional protein classes, including structural, cytoskeletal, and redox-related proteins such as CYB5R3, highlighting the complementary insights provided by different proteomics technologies.

Moreover, unlike the recent microvesicle-focused proteomic study in pediatric Fontan patients with advanced cardiac dysfunction [9], our serum-based approach captures systemic proteomic alterations across a broader clinical spectrum and differing risk profiles. Importantly, distinct proteomic patterns differentiated patients according to risk profile, suggesting a shift from active ECM remodeling toward a more established fibrotic state in individuals with a less favorable profile. Beyond these overarching patterns, individual proteins, notably CYB5R3 and EMILIN1, may serve as candidate biomarkers to guide risk stratification and targeted intervention.

### 3.1. Fontan vs. Healthy Individuals

The widespread divergence in protein expression (Figure 1) suggests a fundamental shift in the systemic proteomic landscape of individuals with a Fontan circulation. The pronounced molecular differences underscore the pathophysiological complexity of the Fontan state and highlight the extent of systemic remodeling, likely reflecting hemodynamic stress, metabolic dysregulation, and chronic inflammation. While this strong separation between patients and healthy controls validates the biological relevance of the proteomic signal, it also illustrates the challenge of identifying disease-specific biomarkers due to the diffuse and multifactorial nature of the observed changes. However, the cluster analysis (Figure 2) highlights key biological systems and indicates how they respond differently in Fontan patients.

Of the 23 proteins grouped by the ECM cluster, 21 had a higher abundance in patients. Enhanced expression of ECM proteins is particularly relevant in the context of angiogenesis and the development of collateral circulation, both hallmarks of long-term adaptation in Fontan physiology [11]. In a similar independent study, Kelly et al. already highlighted the significance of hypoxia for proteomic alterations. This may explain the observed elevation of proteins involved in angiogenesis (ANGPT2) and progressive fibrosis, with chronic inflammation possibly involved as well [7]. Chronic hypoxia has been shown to stimulate ECM gene expression, while sustained inflammation promotes pathways regulating fibrogenesis through activation of fibroblasts and increased deposition of matrix components such as collagens and fibronectin [12,13]. These alterations contribute to fibrotic stiffening, vascular remodeling, and potentially impaired organ function over time.

In the ACO cluster all 27 proteins showed a lower abundance in the patient cohort compared to controls. This may reflect multiple converging pathophysiological influences characteristic of the Fontan circulation. Chronic low-grade inflammation, common in individuals with a Fontan circulation, is associated with elevated levels of pro-inflammatory cytokines such as TNF-α, IL-6, and interferon-γ. These mediators are known to suppress the expression of structural cytoskeletal proteins and may contribute to endothelial dysfunction and compromised cellular integrity [14]. In parallel, PLE and increased CVP can lead to lymphatic congestion and impaired transport of immune mediators and nutrients, conditions that further destabilize cellular homeostasis [15,16]. Furthermore, chronic hypoxia and restricted cardiac output may induce an energy-conserving state in diverse tissues, wherein energetically demanding structural proteins are downregulated. In cardiomyocytes, reduced expression of actin-associated proteins may impair mechanical stability, excitation, contraction coupling, and intracellular signaling, contributing to decreased myocardial contractility, disorganization of cardiac structure, and progression toward heart failure [17]. Additionally, the combined reduction in ACO and PAPs, along with previously reported changes in erythrocyte deformability and aggregation, points to an adaptive remodeling of cellular mechanics in response to the low-shear, non-pulsatile flow of the Fontan circulation [18].

With a focus on PAPs, 34 out of 37 proteins in this cluster had a lower abundance in Fontan patients compared to controls. These proteins included integrin-mediated signaling components (e.g., ITGB3, TLN1, FERMT3) and proteins involved in secretion and activation cascades (e.g., RAP1B, MYH9, RAB27B). This reduction may represent an adaptation to the prothrombotic, low-flow environment of the Fontan circulation and is likely influenced by the widespread use of anticoagulation, reflecting the current clinical management of this patient cohort. In line with these findings, previous studies have reported mild acquired von Willebrand syndrome and platelet dysfunction in Fontan patients, supporting the concept of a secondary adaptation of hemostasis to chronic venous stasis and anticoagulant therapy [19].

### 3.2. Single Case Observation

The markedly increased EMILIN1 abundance observed in patient 01 (FC = 14.4) without anticoagulant or antiplatelet therapy may indicate modulation of TGF-β-related pathways: EMILIN1 is an ECM- and platelet-associated glycoprotein which regulates vascular structure and extracellular matrix organization by binding to pro–TGF-β and preventing its activation, thereby limiting profibrotic signaling [20]. Even if this finding of elevated EMILIN1 in this single patient should be interpreted as a case study rather than a generalizable cohort effect, it raises the possibility of an interaction between anticoagulation therapy and ECM/platelet signaling. Both vitamin K antagonists and aspirin modulate platelet activity and downstream signaling, which can influence the secretion or stability of ECM proteins. EMILIN1, a key component of elastic fibers and a modulator of TGF-β signaling, and thus a key regulator of elastogenesis, vascular integrity, and integrin-mediated cell adhesion may be affected by these pathways, potentially explaining its elevation in the non-anticoagulated patient. Although speculative, this observation suggests that anticoagulation could attenuate circulating ECM protein levels, highlighting an intriguing basis for further investigation in larger cohorts of Fontan patients [21]. This observation is based on a single non-anticoagulated patient and should therefore be interpreted as exploratory. Patient 01 can be viewed as a biological positive control, revealing platelet-associated protein patterns in the Fontan circulation without pharmacological interference.

### 3.3. Classification with Respect to Fontan Status

In the comparison between patients with a favorable and an unfavorable Fontan profile, CYB5R3 was the only protein reaching statistical significance, contrasting with the pronounced differences seen between patients and healthy controls. This limited intra-cohort divergence illustrates the biological complexity of Fontan physiology and highlights a potential role in the metabolic adaptation to chronic circulatory stress in that subgroup of patients. CYB5R3 is a central regulator of redox homeostasis, nitric oxide metabolism [22], and cellular aging [23]. As a reductase, CYB5R3 influences lipid metabolism and cholesterol biosynthesis while reducing heme iron and oxidized co-enzyme Q to quench reactive oxygen species (ROS). CYB5R3 reduction protects against lipid peroxidation, a process in which ROS damage cell membranes. This study found that CYB5R3 was elevated in Fontan patients with unfavorable hemodynamics, possibly indicating heightened oxidative stress or endothelial dysfunction. Kelly et al.’s proteomics study recently found downregulation in superoxide dismutase activity and elevated levels of enzymes related to production, cleaving, and recycling glutathione [7]. Its elevation in abundance may result from chronic hypoxia and inflammation, which induce redox-regulatory enzymes such as the hypoxia-induced factor or the nuclear factor kappa-light-chain-enhancer of activated B-cells. In an animal model, CYB5R3 in endothelial smooth muscle cells was found to protect cardiac and vascular function under chronic hypoxic stress [24]. CYB5R3 transfers electrons from nicotinamide adenine dinucleotide hydrogen to cytochrome b_5_, a heme-iron–containing protein, making its function dependent on iron availability [25]. Functional activity may remain limited due to iron deficiency [26,27], frequently observed in individuals with a Fontan circulation, representing a classic adaptive but incomplete redox response. Previous studies support the concept of dysregulated oxidative balance in Fontan patients. Elevated asymmetric dimethylarginine and methionine-sulfoxide have been documented through targeted metabolomics [28], and proteomic analyses revealed a signature of oxidative stress and apoptosis [6,7,9]. In a serum lipidomics analysis, elevation of phosphatidylserines was considered as caused by apoptosis, oxidative stress and inflammation [29]. Moreover, accelerated epigenetic aging has been reported both in children and adults with a Fontan circulation, suggesting sustained cellular stress [30]. Evaluating methemoglobin levels in patients with more pronounced redox balance may help to clarify whether elevated CYB5R3 expression reflects an impaired capacity to maintain redox homeostasis or a compensatory response to chronic oxidative pressure. Taken together, CYB5R3 may represent a component of a candidate biomarker panel for Fontan phenotyping and risk stratification, capturing adaptive metabolic and redox processes in response to the unique hemodynamic burden of Fontan physiology.

### 3.4. Subgroup Analysis with GSEA

In comparisons with their healthy controls, HEMT-related proteins were consistently enriched in Fontan patients, indicating ongoing matrix remodeling. Within the Fontan cohort, however, HEMT enrichment was lower in patients with reduced EF or an increased risk score, which may reflect a transition from active remodeling to a more established fibrotic state where signaling diminishes despite persistent structural changes. In contrast, the GOCC cytosol gene set was globally reduced in the Fontan cohort compared to controls but showed higher enrichment in unfavorable subgroups such as patients with a risk factor > 3 or a morphological RV. This pattern may point to increased cytosolic protein release under adverse hemodynamic conditions, possibly reflecting endothelial or hepatic stress. Taken together, these opposing trends suggest that individuals with a Fontan circulation undergo ECM-driven remodeling at large, while disease severity within the cohort is characterized by reduced HEMT activity alongside increased stress-related cytosolic signatures, warranting validation in larger patient groups.

### 3.5. Limitations

The analysis was constrained by a limited number of participants, restricting the available dataset for group comparisons. Further subdivision within the Fontan cohort, such as unfavorable versus favorable profile, ventricular morphology, EF, or risk classification, reduced the number of observations per subgroup, thereby limiting statistical power. Incomplete availability of variables contributing to the composite risk score may have introduced bias, potentially leading to misclassification of patients into the favorable risk category. The study’s cross-sectional design, encompassing patients with various configurations of the Fontan circulation, precludes conclusions about longitudinal or adaptive changes, underscoring the need for follow-up investigations. Sample collection was confined to peripheral venous blood. Although samples were collected under standardized fasting conditions and individually age- and sex-matched to healthy controls, potential (subclinical) protein loss cannot be excluded as a confounding factor in the analysis of systemic blood proteins. Examination of samples from different compartments, such as pulmonary vs. Fontan tunnel blood, lymphatic fluid from various sites, or liver and myocardial tissue, as well as larger, specific subgroups, would be informative. A multicenter approach will be essential to overcome these limitations. Methodological considerations related to the chosen quantitative proteomics strategy should therefore also be acknowledged. One limitation of isobaric labeling–based quantification is the high cost of reagents. However, while label-free approaches may be more economical for small sample numbers, the multiplexing capability of isobaric labeling (e.g., TMT/TMTpro) can offset these costs in large-scale studies by reducing MS time per sample and increasing throughput. Moreover, label-free methods often require additional technical replicates to achieve comparable statistical power, further increasing instrument usage costs [31]. Finally, the absence of genomic data adds unmeasured heterogeneity, limiting the identification of subtype-specific proteomic signatures. Importantly, by focusing on network-level and stratified analyses by targeted pathway enrichment, the present study provides a structured foundation, paving the way for future comprehensive and cross-platform explorations of Fontan-associated proteomic alterations.

## 4. Materials and Methods

### 4.1. Study Population

A prospective, cross-sectional investigation was conducted involving a total of 48 participants, encompassing pediatric, adolescent, and adult individuals (aged 4–38 years) with a completed Fontan circulation. Of the initially enrolled 54 Fontan patients, 48 had sufficient serum material available for proteomic analyses and were therefore included in the final study cohort. Patients were recruited from a specialized center for congenital heart disease, the Ruhr University Bochum (Bochum, Germany). This cohort represented a broad spectrum of Fontan modifications, including both lateral tunnel and extracardiac conduit configurations, with some participants having undergone surgical revisions or prior aortic procedures. To enable comparative analysis, a control group of individually age- and sex-matched healthy volunteers with normal biventricular cardiac anatomy was assembled from a second university center (Medical University of Innsbruck, Innsbruck, Austria).

Each subject attended a standardized assessment visit, including detailed medical history, physical examination, and documentation of clinical data such as prior surgeries, time since Fontan completion, and current pharmacotherapy. Routine laboratory testing was performed. Additional investigations such as transthoracic echocardiography, cardiac magnetic resonance imaging, treadmill exercise testing, and liver sonography were conducted in some patients, as summarized in Table 1. For the proteomics serum analyses, all participants were fasting overnight at the time of venous blood draw. Within 20 min after blood draw, serum was processed by immediate centrifugation and stored at −80 °C until proteomic profiling. To ensure data integrity and reduce variability, all steps of sample collection and processing adhered to standardized protocols [32]. The study was conducted in accordance with the Declaration of Helsinki and approved by the local ethics committees, and written informed consent was obtained from all participants or their legal guardians.

### 4.2. Clinical Classification

To discriminate between individuals with a favorable or unfavorable Fontan profile, a 12-parameter score based on previously published risk scoring systems [33,34] was applied in the present study (Supplementary Data S1). Minor modifications, such as a lower cut-off threshold, were implemented to enable clearer discrimination within the study cohort. For each patient, one point was assigned for the presence of any of the following 12 parameters: open fenestration, tunnel dilation, PLE, elevated central venous pressure (CVP) (>15 mmHg), reduced oxygen saturation (<90%), significant collateral flow on angiography, diaphragmatic paresis, decreased exercise capacity (peak oxygen consumption < 45% of the predicted value [35]), morphologically systemic right ventricle (RV), significant valve regurgitation, reduced ejection fraction (EF) (<50%), and a cardiac index < 2.5 L/min/m^2^. Each parameter was scored as 1 if present and 0 if absent, yielding a total score ranging from 0 to 12. Patients with a score of 3 or less were categorized as having a favorable Fontan profile, while those with more than 3 points were considered to have an unfavorable Fontan profile, reflecting more concerning hemodynamics.

### 4.3. Sample Processing and LC-MS/MS

For proteomic profiling, serum samples from Fontan patients and matched healthy controls were prepared using a standardized workflow. Each individual patient sample constituted a biological replicate. No technical replicates were performed. High-abundance serum proteins such as albumin and immunoglobulins were selectively depleted (High Select™ Depletion Spin Columns, Thermo Fisher Scientific, Vienna, Austria) to increase the dynamic range for low-abundance proteins. The performance of single-use spin columns for human plasma depletion has been evaluated by Cao et al. [36], demonstrating that High Select™ Depletion Spin Columns provide a reproducible and cost-effective approach for high-abundance protein removal prior to in-depth MS-based plasma analysis. The remaining protein fraction was enzymatically digested with trypsin (Sequencing Grade Modified Trypsin, Promega, Walldorf, Germany) into peptides. These peptides were subsequently labeled using Tandem Mass Tag (TMT) reagents (TMT10plex™ Label Reagent Set, Thermo Fisher Scientific, Vienna, Austria) to allow for relative quantification across samples. Labeled peptides were pooled, fractionated, and analyzed by nano-liquid chromatography (UltiMate 3000 RSLCnano system, Thermo Fisher Scientific, Vienna, Austria) coupled to a high-resolution Orbitrap-based mass spectrometer (Orbitrap Eclipse, Thermo Fisher Scientific, Vienna, Austria) operating in data-dependent acquisition mode [37,38].

### 4.4. Quantification and Normalization

Peptides were identified by database searching against a curated human protein reference set using ProteomeDiscoverer 3.1 software (Thermo Fisher Scientific). Quantitative information was derived from the intensity of TMT reporter ions. To ensure comparability across samples, data were normalized to account for batch effects and technical variation. All samples passed quality control thresholds for peptide recovery and labeling efficiency. The dataset comprised 6 pools of 16 samples each, along with two internal standards. Proteins that were detected in only two or fewer of the 6 batches were excluded from further analysis. All proteins identified and used for downstream analysis are provided in Supplementary Data S2.

### 4.5. Thresholds for Significance

Statistical comparisons between groups (Fontan vs. control and between Fontan subgroups) were conducted to detect proteins with differential expression. Abundance ratio/fold change values were calculated for all identified proteins, and statistical significance was determined using moderated *t*-tests with correction for multiple hypothesis testing per comparison via the Benjamini–Hochberg method [39]. For group-level comparisons (Fontan vs. control), FC values were calculated relative to the corresponding controls. For subgroup analyses within the Fontan cohort (e.g., left vs. right ventricular morphology, favorable vs. unfavorable Fontan profile), FC values were derived from direct comparisons between the respective patient subgroups without inclusion of the control group. Proteins were considered significantly altered in abundance if they met an adjusted *p*-value (FDR) < 0.05 and a FC > 1.5 or <0.67.

### 4.6. Search Tool for Retrieval of Interacting Genes/Proteins Analysis

STRING (search tool for retrieval of interacting genes/proteins) network analysis was performed to explore potential functional relationships among the differentially abundant proteins (Fontan vs. control) using the subset of proteins that showed significant differences between Fontan patients and healthy controls. Of the 2288 proteins initially identified, 124 met the criteria defined in this study for differential abundance and were selected for further analysis using the STRING database (https://cn.string-db.org/, accessed on 18 January 2026). The resulting interaction networks were evaluated based on the strength and confidence of predicted associations, as indicated by STRING internal scoring metrics such as “strength” and “signal,” which reflect the relevance and robustness of the protein–protein functional or physical associations within the network.

### 4.7. Volcano Plots for Visualizing Differential Expression

Volcano plots were generated to visualize the distribution of differentially abundant proteins across key group comparisons. Proteins meeting significance and FC thresholds were color-coded to indicate increased or decreased abundance, enabling intuitive assessment of global expression changes.

### 4.8. Gene Set Enrichment Analysis

GSEA was performed to identify pathway-level changes associated with the observed proteomic alterations. HEMT and GOCC gene sets were selected to capture key molecular mechanisms in Fontan physiology, with HEMT reflecting extracellular matrix remodeling and fibrotic activity, and GOCC indicating cellular stress and altered compartment integrity. Comparisons included Fontan vs. control, favorable vs. control, unfavorable vs. control, and additional clinical subgroups such as EF (low vs. high), risk score ≤ 3 vs. >3 (favorable vs. unfavorable), ventricular morphology, etc. Enrichment was evaluated using NES and FDR-adjusted *p*-values (<0.05). The NES indicates whether a gene set is predominantly up- or downregulated in one group relative to the other, normalized for gene set size. This approach allowed for the identification of systematically elevated or reduced biological processes across larger groups, complementing individual protein-level analyses [40].

## 5. Conclusions

This analysis demonstrates extensive systemic protein remodeling in patients with a Fontan circulation, particularly in pathways related to ECM regulation, ACO, and PAPs. Using an unbiased proteomic approach, our study extends beyond prior published antibody-based panels focused on adult cohorts and inflammatory or angiogenic markers.

Differences between patient subgroups were subtler than those versus controls but revealed distinct patterns associated with unfavorable risk profiles. Altered ECM proteins and EMILIN1 suggest a shift from active remodeling toward established fibrosis, implicating TGF-β-mediated processes, whereas elevated CYB5R3 reflects disruptions in redox homeostasis, nitric oxide metabolism, and cellular aging, independent of fibrotic remodeling. While comparisons with controls underscore the biological impact of Fontan physiology, intra-cohort signals reflect disease complexity and challenges for clinical translation. Longitudinal and multicenter studies are needed to validate the pathways and clarify their utility for phenotyping, timely diagnosis, risk stratification, and therapeutic targeting.

## Figures and Tables

**Table 1:** Overview of participant characteristics and composite Fontan risk score parameters.

Items	Unit	N	Patients	Controls
Age, mean (SD)	years	48	18.4 (7.6)	18.5 (7.6)
Female sex, n (%)		48	21 (44)	21 (44)
Weight, mean (SD)	kg	48	52.7 (19)	58.3 (17.2)
Height, mean (SD)	cm	48	152.5 (29.4)	165.8 (17.7)
BMI, mean (SD)	kg/m^2^	48	21.3 (6.3)	20.62 (3.0)
* Right ventricle, n (%)		48	22 (46)	
* Aortic Valve Regurgitation, n (%)		39	8 (21)	
Time elapsed since TCPC, mean (SD)	years	48	14.9 (7.65)	
* Open fenestration, n (%)		39	7 (18)	
Ejection fraction, mean (SD)	%	48	50 (10)	
* Ejection fraction < 50%, n (%)		48	23 (48)	
Cardiac index, mean (SD)	L/min/m^2^	40	1.95 (0.83)	
* Cardiac index < 2.5 L/min/m^2^, n (%)		39	26 (66)	
Significant AVVR, n (%)		39	8 (21)	
Peak SO_2_, mean (SD)	%	46	93 (4)	
* Peak SO_2_ < 90%, n (%)		46	8 (17)	
Minimal SO_2_ under exercise, mean (SD)	%	27	87 (6)	
* Collateral flow on angiography, n (%)		27	12 (44)	
* Tunnel dilation on angiography, n (%)		48	8 (17)	
* Central venous pressure > 15 mmHg, n (%)		28	0 (0)	
* Diaphragmatic paresis, n (%)		48	0 (0)	
$\overset{\cdot }{V}$O_2_AT, mean (SD)	mL/kg/min	24	24 (6)	
Peak $\overset{\cdot }{V}$O_2_, mean (SD)	mL/kg/min	25	28 (6.6)	
% of predicted peak $\overset{\cdot }{V}$O_2_ ≥ 80, n (%)		25	5 (20)	
% of predicted peak $\overset{\cdot }{V}$O_2_ ≥ 50, n (%)		25	21 (84)	
* Peak $\overset{\cdot }{V}$O_2_ < 45% of normal, n (%)		25	2 (8)	
* Protein losing enteropathy, n (%)		25	5 (20)	
Vitamin K antagonist, n (%)		48	43 (90)	
Acetylsalicylic acid, n (%)		48	4 (8)	
Elastography ≥ 22 kPa, n (%)		20	4 (20)	
METAVIR Score ≥ 4, n (%)		18	11 (61)	
AST, mean (SD)	U/L	28	37 (14)	
ALT, mean (SD)	U/L	29	30 (16)	
AP, mean (SD)	U/L	18	138 (92)	
γGT, mean (SD)	U/L	25	68 (50)	
γGT ≥ 50 [U/L], n (%)		25	14 (56)	
Hemoglobin, mean (SD)	g/dL	36	15 (2)	
Hemoglobin ≥ 15 g/dL, n (%)		36	17 (47)	
Platelets, mean (SD)	1/µL	36	225 (102)	
Platelets ≤ 150,000/µL, n (%)		33	13 (39)	
Total protein, mean (SD)	g/dL	37	6.9 (1)	
Albumin, mean (SD)	g/dL	31	4 (0.7)	
Risk score ≤ 3, n (%)		48	37 (77)	

ALT, alanine transaminase; AP, alkaline phosphatase, AST: aspartate aminotransferase, AT, anaerobic threshold; AVVR, atrioventricular valve regurgitation; BMI: body mass index; γGT: gamma-glutamyl transferase; METAVIR, meta-analysis of histological data in viral hepatitis; N, sample size; SD, standard deviation; SO_2_, (pulse oximetric) oxygen saturation; TCPC, total cavopulmonal connection; $\overset{\cdot }{V}$O_2_, oxygen uptake. *, parameters constituting the 12-parameter risk score. One single patient did neither receive anticoagulation nor antiplatelet therapy. Further medications included ACE inhibitors (n = 11), L-thyroxine (n = 9), and diuretics (n = 7), beta-blockers (n = 6), sildenafil (n = 1), propafenone (n = 1).

**Table 2:** Differentially expressed proteins identified by STRING network analysis grouped into three functional categories.

Actin Cytoskeleton Organisation			Extracellular Matrix			Platelet Function		
Protein	Accession	Fold Change	Protein	Accession	Fold Change	Protein	Accession	Fold Change
FLII	Q13045	0.56	RELN	P78509	1.87	CNN2	Q99439	0.57
WDR1	Q75083	0.66	EMILIN1	Q9Y6C2	1.81	TPM4	P67936	0.57
ARAP1	Q96P48	0.60	FGB	P02675	1.72	CAP1	Q01518	0.65
NCK2	O43639	0.61	THBS2	P35442	1.80	DNM1L	O00429	0.62
PLEK	P08567	0.54	AEBP1	Q8IUX7	2.78	GAPDH	P04406	0.61
NCKAP1L	P55160	0.66	ANGPT2	O15123	1.83	MYH9	P35579	0.64
PARVB	Q9HBI1	0.61	FBLN2	P98095	1.66	CD9	P21926	0.66
TLN1	Q9Y490	0.60	ADIPOQ	Q15848	1.62	FERMT3	Q86UX7	0.63
ROCK2	O75116	0.62	CDH2	P19022	1.56	LASP1	Q14847	0.62
ARPC1B	O15143	0.62	LTBP2	Q14767	1.73	EMILIN1	Q9Y6C2	1.81
ZYX	Q15942	0.61	DCN	P07585	1.71	NCK2	O43639	0.61
ACTR3	P61158	0.66	FBLN5	Q9UBX5	1.67	RAP1B	P61224	0.55
FLNA	P21333	0.59	ADAMTSL2	Q86TH1	2.32	GP1BB	P13224	0.63
TPM3	P06753	0.63	LTBP4	Q8N2S1	1.68	DOK1	Q99704	0.50
ACTN1	P12814	0.61	PODN	Q7Z5L7	1.59	FGB	P02675	1.72
PDLIM1	O00151	0.53	IGFBP7	Q16270	1.70	FGG	P02679	1.93
CNN2	Q99439	0.57	FGG	P02679	1.93	ITGB3	P05106	0.66
CORO1C	Q9ULV4	0.65	CTSB	P07858	1.55	LIMS1	P48059	0.64
CSRP1	P21291	0.61	SERPINB1	P30740	0.57	ARPC1B	O15143	0.62
TPM4	P67936	0.57	A2M	P01023	1.59	ZYX	Q15942	0.61
DSTN	P60981	0.64	CLC	Q05315	0.64	TLN1	Q9Y490	0.60
TPM1	P09493	0.64	CBLN4	Q9NTU7	1.80	TPM3	P06753	0.63
CAP1	Q01518	0.65	COL6A1	P12109	1.54	COTL1	Q14019	0.62
TMSB4X	P62328	0.48				TAGLN2	P37802	0.59
CFL1	P23528	0.58				ACTN1	P12814	0.61
PFN1	P07737	0.56				FLNA	P21333	0.59
MYH9	P35579	0.64				CFL1	P23528	0.58
						PDLIM1	O00151	0.53
						FLII	Q13045	0.56
						YARS1	P54577	0.61
						SBDS	Q9Y3A5	0.63
						WDR1	O75083	0.66
						ATP2A3	Q93084	0.63
						SERPINB1	P30740	0.57
						RAB27B	O00194	0.62
						LY6G6F	Q5SQ64	0.57
						NCF1B	P02679	0.57

The three categories are: actin cytoskeleton organization (ACO), extracellular matrix (ECM) remodeling, and platelet-associated proteins (PAPs). For each protein the UniProt accession number and fold change (FC) are listed. All ACO associated proteins showed reduced abundance in Fontan patients compared to controls, while ECM proteins were predominantly upregulated. 33 out of 37 proteins within the PAPs cluster exhibited a reduced FC.

## Data Availability

The original contributions presented in this study are included in the article/Supplementary Material. Further inquiries can be directed to the corresponding author.

## Supplementary Materials

The following supporting information can be downloaded at: https://www.mdpi.com/article/10.3390/ijms27031220/s1

## Abbreviations

The following abbreviations are used in this manuscript:

ACO	Actin cytoskeleton organization
CVP	Central venous pressure
CYB5R3	Cytochrome b5-reductase 3
ECM	Extracellular matrix
EF	Ejection fraction
EMILIN1	Elastic microfibril interface located protein-1
FC	Fold change
GOCC	Gene Ontology Cellular Component
GSEA	Gene set enrichment analysis
HEMT	Hallmark epithelial–mesenchymal transition
LV	Left ventricle
NES	Normalized enrichment score
NYHA	New York heart association
PAPs	Platelet-associated proteins
PLE	Protein-losing enteropathy
ROS	Reactive oxygen species
RV	Right ventricle
STRING	Search tool for retrieval of interacting genes/proteins
SVHD	Single-ventricle heart disease
TCPC	Total cavopulmonary connection
TGF-β	Transforming growth factor–β
TMT	Tandem Mass Tag
